# Research note: The effect of electron-beam irradiation on microbial load, shelf-life and color of refrigerated chicken tenders

**DOI:** 10.1016/j.psj.2026.107093

**Published:** 2026-05-07

**Authors:** Pheron Collie, Blesseth McDonald, Olivia Hawkins, Marco Reina, Andrew Widmer, Gabriela K. Betancourt-Barszcz, Aaron R. Bodie

**Affiliations:** a University of Georgia, Department of Poultry Science, Athens, Georgia 30602; b Food Product Innovation & Commercialization Center, Griffin, Georgia 30223; c Reveam, Inc. Norcross, Georgia 30071

**Keywords:** Color, E-beam, Spoilage, Storage, Tenderloins

## Abstract

Electron-beam (E-beam) irradiation is a non-thermal intervention with the potential to reduce microbial load and extend shelf-life in raw poultry products. The objective of this study was to evaluate the effectiveness of E-beam irradiation doses on microbial load, color stability, and shelf-life parameters of tray packed chicken tenders stored under refrigerator conditions for 27 days. Across all sampling days, E-beam irradiation significantly reduced APC compared to the nonirradiated control (p < 0.05), with suppression of microbial growth persisting throughout refrigerated storage. By day 27, irradiated samples exhibited reductions of 2.90 to 5.90 Log CFU/g compared to the control. Enterobacteriaceae (EB) was largely undetectable in irradiated samples during early storage. At day 27, irradiated samples were significantly lower than control samples, indicating delay in microbial growth. E-beam irradiation consistently increased redness(a*) across storage days independent of dose, while changes in lightness (L*) and yellowness (b*) were minimal, time-dependent and largely remained within acceptable ranges for poultry meat. Ranges 1.5 and 2.5 kGy provided effective microbial suppression with minor impact on color. These findings demonstrate that E-beam irradiation has the ability to alter spoilage of refrigerated chicken tenders by reducing initial microbial load and delaying the onset of microbial growth, while maintaining acceptable color characteristics.

## Introduction

Increased demand for poultry meat has challenged the poultry industry to ensure product safety and extend the shelf-life of poultry products for consumers. One of the main hurdles to achieving these goals is that poultry is a natural reservoir for foodborne pathogens such as *Salmonella* and *Campylobacter* (Chai et al., 2017). Compounding this issue, raw poultry meat typically has a refrigerated shelf-life of only about two days following purchase from retail (United States Department of Agriculture-Food Safety and Inspection Services, 2024; USDA-FSIS). The growth of spoilage bacteria reduces product quality and acceptability, due to the development of rancid off-odors and changes in color and taste of poultry products. To address this issue, processing plants typically apply antimicrobial interventions, such as peracetic acid (PAA), and chlorine (Chapagain et al., 2026). However, the effects are short term, only reducing initial bacterial load. As a result, the poultry industry continues to seek novel interventions that enhance food safety and extend the shelf-life of raw poultry meat.

One promising intervention is the use of electron-beam (**E-beam**) irradiation, which is a non-thermal sterilization process that applies ionizing energy to the desired food product. This irradiation method delivers a stream of high-energy electrons that penetrate bacterial cells, causing DNA cleavage and cellular damage, thereby rendering the bacteria non-viable. The use of E-beam irradiation could offer various advantages over other irradiation methods approved for food products (like gamma-rays and X-rays) (Morehouse and Komolprasert, 2018). For example, when compared to gamma rays, E-beam does not use radioactive sources (like Cobalt-60 or Cesium-137). Another advantage is that E-beam can be easily turned on and off as needed (Morehouse and Komolprasert, 2018). Moreover, E-beam is more efficient than X-ray irradiation, as the conversion of electrons to X-rays is inefficient (typically only 8–12%) and requires more electricity to achieve equivalent lethality (Morehouse and Komolprasert, 2018). Such advantages make E-beam treatment an attractive option for processors seeking an intervention to improve poultry food safety and product longevity.

Prior E-beam studies have largely focused on evaluating immediate post-treatment reductions. While this portrays E-beam reductions effectively, it does not account for post-treatment microbial growth, spoilage kinetics and quality throughout shelf-life. Such information is critical for poultry processing plants to determine optimal dose levels necessary for microbial control and meat quality parameters. To address this, the goal of this study was to investigate how E-beam doses influence microbial load, color stability, and shelf-life of refrigerated chicken tenders over 27 days of storage.

## Materials and methods

### Meat samples

For this study, fresh tenderloins were used immediately following processing. All meat samples were collected directly after processing from a commercial poultry processing facility (USA). Boneless, skinless tenderloins were tray packed in 1.9-pound packs. Packaged meat samples were placed on ice in a Styrofoam cooler and maintained at refrigerated temperatures between 0 and 4°C until they were subjected to E-beam irradiation treatments. Transportation of chicken took 24 hours to reach the irradiation treatment facility.

### E-beam treatment application

Once samples arrived at the irradiation facility, they were aseptically removed from the Styrofoam cooler. All samples were placed on the conveyor and passed through the E-beam system. The E-beam application was done while tenderloins were inside the tray pack bundles. Treatments used for this study were no-treatment control, 1 kGy, 1.5 kGy, 2.5 kGy and 3.5 kGy (Morehouse and Komolprasert, 2018). All meat samples were treated within their tray packaged packs. Treated samples were one-day shipped to the University of Georgia poultry science department, temperature maintained between 0 and 4°C. Upon arrival at the University of Georgia, all samples were placed in a walk-in cooler with temperatures maintained at 4°C and kept until their respective sampling time point.

### Microbial analysis

There were five technical replicates for each treatment group per timepoint. At each timepoint, E-beam treated samples were removed from the refrigerator and transferred to the BSL 2 lab for microbial analysis. Tenderloins were aseptically removed from tray packs and 50 g were weighed into sterile sample Whirl-Pak bags (Whirl-Pak, Wisconsin, USA). Simultaneously, 400 mL of Buffered Peptone water (BPW) (Hardy Diagnostics, California, USA) were aliquoted into each bag. Samples were stomached (Seward Stomacher Model 400; Seward Laboratory, New York, USA) for 2 minutes at 230 rpm, and the resulting rinsates were collected for downstream analysis. E-beam treated tenderloin rinsates were then aliquoted into 10 mL test tubes. Serial dilutions were prepared to 10^-7^ using 1:10 serial dilutions in BPW. Following rinsate dilutions, 1 mL from each dilution was plated on Aerobic Plate Count (APC) (3 M, Minnesota, USA) and Enterobacteriaceae (**EB**; 3 M, Minnesota, USA) Petrifilm in duplicates. All plates were incubated overnight (18–24 hours) at 37°C. All counts were Log transformed into CFU/g using the formula:

$$CFU/mL=\frac{\;\text{Colonies}\text{counted}\;}{\;\text{Dilution}\text{factor}\;}$$

### Colorimeter

On the day of sampling, three individual tenderloins were sampled for color from each treatment replicate. Samples color was quantified using the CIE L*a*b* system, a widely adopted framework for objective meat color evaluation. In this color scale, L* represents lightness, whereas a* and b* describe chromaticity along the red–green and yellow–blue axes, respectively. Greater L* values indicate a lighter appearance, while positive a* and b* values correspond to increased redness and yellowness. Color data were measured using a colorimeter (CR-10 Plus, Konica Minolta, Osaka, Japan). Measurements were taken at three locations on each tenderloin to account for surface variability. The instrument was calibrated before each use with standardized white tiles enclosed in a polyethylene barrier. Each location was measured in duplicates, and paired readings were averaged prior to statistical analysis.

### Statistical analysis

All analyses were performed using log₁₀-transformed CFU/g values. APC and EB data were analyzed separately for each storage day using a one-way ANOVA with treatment as the fixed effect. Technical replicates (n = 5 per treatment) were treated as independent observations. Means were separated using Tukey’s HSD at α = 0.05. For color analysis, L*, a*, and b* were compared each day respectively using a one-way ANOVA followed by a Tukey’s HSD. All statistics were done using R 4.5.2.

## Results and discussion

### APC and EB

**Fig. 1:**
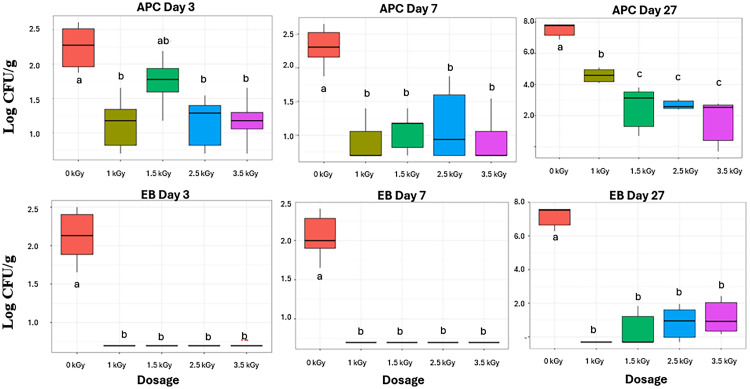
Box plots of E-beam treated chicken tender microbial Log CFU/g plate counts. The X-axis indicates E-beam Irradiation dosage (kGy) while Y-axis indicate Log CFU/g. Top: Total Aerobic plate counts for days 3, 7 and 27. Bottom: Total Enterobacteriaceae counts for days 3, 7 and 27. ^a- c^ Means with different letters within a storage day and color type are significantly different (*p* < 0.05).

In poultry production, aerobic bacteria are used as a general indicator for overall bacteria load and process control. Spikes in APC counts are associated with low performance interventions, which lead to faster spoilage in the final product. The effect of the treatments was analyzed by day to determine the efficacy of the irradiation, on the growth potential of aerobic bacteria on day 3, 7 and 27, independently (Fig. 1). Throughout the 27 days, ANOVA revealed a significant difference between all treatments on each day (p < 0.05). On day 3, treatments 1 kGy (1.23 Log CFU/g), 2.5 kGy (1.34 Log CFU/g) and 3.5 kGy (1.21 Log CFU/g) showed a significant difference for APC microbial load compared to the control that was at least 0.6 Log CFU/g higher (2.23 Log CFU/g; p < 0.01), whereas 1.5 kGy (1.68 Log CFU/g) did not differ significantly. By day 7, we observed significant reduction in E-beam treatments ranging from 1.18 through 1.44 log CFU/g as compared to the control group (p < 0.01). The control group had 2.29 Log CFU/g. All irradiated treatments were significantly different from the control group; however, between 1 kGy through 3.5 kGY, treatments were statistically the same. Previously, Kalapala et al. (2025) highlighted that electron beam application can reduce aerobic bacterial load on chicken and turkey meat significantly. The authors observed that the E-beam treatment reduced total aerobic bacterial counts from 8.12 Log CFU/g in the untreated control samples to 5.82 CFU/g at 1 kGy, and 3.45 CFU/g at 1.5 kGy. The higher treatment dose (2.5 and 3.5 kGy) was below detection. Consequently, our results indicate E-beam reduces aerobic bacteria on chicken tenders and suppresses growth over time, compared to not treating with E-beam.

By day 27, aerobic bacteria reduction was observed from 2.90 to 5.90 Log CFU/g compared to the control group. The control group had the highest recovered bacteria concentration of 7.52 Log CFU/g. These results demonstrate a dose-response relationship between the concentration of the E-beam and aerobic bacteria. Additionally, the number of days in storage also significantly affected the number of bacteria recovered from treated chicken tenders. However, all treatments were significantly lower than the non-treated control treatment, suggesting that regardless of storage time, E-beam is effective at reducing overall bacteria load. The suppressed APC levels in our results indicate that E-beam treatment disrupts this storage trajectory by delaying growth.

EB is usually associated with the presence of gut-related bacteria. While several genera from this family do not pose a risk to human consumption, it includes foodborne pathogens such as *Salmonella, Shigella*, and pathogenic *Escherichia coli*. In the poultry industry, EB plate counts are used as an indicator of the potential for foodborne pathogens. On Day 3 and 7, no treatment group had any growth for colonies; however, control groups grew to 2.10 (Day 3) and 2.04 (Day 7) Log CFU/g, respectively. The most microbial growth was seen on Day 27 of storage. Control treatment was significantly different from all other treatments with 7 Log CFU/g (p < 0.05; Fig. 1). No growth was observed for the 1 kGy treatment; however, this may be attributable to a sampling error as stronger E-beam doses had growth observed. Treatments 1.5 kGy through 3.5 kGy are all less than 1.1 Log CFU/g. These results suggest a dose response relationship between EB bacteria and strength of E-beam applied. Similar results were seen by Kalapala et al., 2025, where they demonstrated no growth for E-beam treated ground chicken at 2 and 3 kGY (Kalapala et. al, 2025).

The absence of EB growth in irradiated samples during early storage suggests that E-beam treatment not only reduces initial contamination but may also delay the onset of microbial rebound. This phenomenon is consistent with the ability of irradiation to disrupt the cellular repair mechanisms in gram-negative bacteria. The divergence between the control and treated samples by Day 27 further indicates that higher doses impede the typical spoilage trajectory of EB.

### Color

When evaluating fresh meat, consumers primarily rely on visual cues, with color exerting the strongest influence on purchase decisions, as texture and odor cannot be assessed until the package is opened (Wideman et al., 2016). Acceptable color often serves as a proxy for overall quality, leading consumers to assume that other attributes such as texture and flavor are also satisfactory (Wideman et al., 2016). Color was assessed to evaluate the effect of E-beam and time on chicken tenders. On Days 3 and 7, irradiated samples exhibited significantly higher a* values compared with the non-irradiated control (p < 0.01), regardless of dose (1 through 3.5 kGy). While numerical differences among irradiation doses were observed, none differed statistically, indicating that the development of a subtle, pink hue on the tenders was driven primarily by irradiation exposure no matter the dosage. The persistent elevation in redness (a*) aligns with several research studies that suggest irradiation induces pinking in poultry meat (Du et al., 2001). This can be primarily attributed to the formation of stabilized myoglobin and partial reduction of ferric heme pigments to ferrous forms that stabilize red coloration.

Lightness (L*) and yellowness (b*) were less responsive and more time dependent. No differences in L* or b* were detected on Day 3 (p > 0.05); however, by Day 7, higher irradiation doses resulted in reduced b* values (6.41–7.21) compared with the control (9.25; p < 0.01). This loss of yellowness is consistent with carotenoid bleaching and oxidative degradation reported previously in irradiated poultry meat (Ahn et al., 1998). By contrast, L* values remained unaffected at Day 7 (p > 0.05), suggesting that irradiation did not alter surface reflectance or moisture related light scattering.

By Day 27, control samples were significantly lighter (L* = 63.90) than irradiated samples (58.92–61.33; p < 0.05). The Lightness (L*) values were interpreted using established classification criteria for the lightness of poultry meat, as it is classified by the category: dark (L* < 55.91), normal (55.91 ≤ L* ≤ 61.55), and pale (L* > 61.55). Our study indicated that from day 3 to day 27, most of the L* data were considered normal according to the criteria; only the control of day 27 filled the pale category. This shift may reflect cumulative pigment oxidation and structural changes in myofibrillar proteins that reduce light reflectance over time (Kaewthong et al., 2019). The 1.5 and 2.5 kGy treatments exhibited higher a* values (2.69–2.92) than the control and 1 kGy samples (1.00–1.21, respectively; p < 0.01), suggesting that irradiation-induced pigment stability is maintained during extended storage. Lastly, no differences in b* were detected, supporting a reversal in yellowness, as treatments 1.5, 2.5 and 3.5 kGy are higher than the control, and 1.0 kGy was lower than the control. This indicates that over time, yellowness may have a dose threshold that stays in an acceptable range more consistent than chicken tenders not treated using E-beam. Similar results were seen with variation over time, for b*, though it fluctuates with E-beam treatments (Wang et al., 2025).

Collectively, these results indicate that redness (a*) is the most robust and stable color indicator of irradiation effects, persisting from early through late storage, whereas changes in lightness and yellowness are weaker, transient, and dependent on both dose and storage duration. These findings reinforce the concept that irradiation driven color changes in poultry are governed primarily by heme pigment chemistry, rather than lipid oxidation or protein denaturation alone, with stable ferrous pigment complexes playing a dominant role in long-term color expression.

E-beam irradiation significantly suppressed aerobic and EB population in chicken tenders through 27 days of refrigerated storage. This indicates sustained disruption of microbial growth rather than short-term load reduction alone. Ultimately, E-beam altered the typical spoilage trajectory, resulting in improved microbial stability over time.

Irradiation increased redness (a*), independent of dose, while changes in lightness and yellowness were modest and remained within acceptable ranges. The findings from this research note suggest that intermediate doses (1.5 to 2.5 kGy) may provide an optimal balance between microbial control and color stability during chicken tender storage. Collectively, this study supports E-beam irradiation as a viable shelf-life intervention for raw poultry products. The next step in this research is to look at specific foodborne pathogens associated with poultry such as *Campylobacter* spp. and *Salmonell*a spp. over an extended storage cycle (Table 1).

**Table 1:** The effect of E-beam irradiation dose and refrigerated storage time on surface color from chicken tenderloin samples over 27 days. ^a^-^b^ Means with different letters within a column per day and color type are significantly different (*p* < 0.05).

Day	Treatment	L*	a*	b*
3	Control	57.15^a^	1.93^b^	9.05^a^
	1 kGy	57.59^a^	4.77^a^	7.02^a^
	1.5 kGy	57.70^a^	4.66^a^	7.27^a^
	2.5 kGy	56.63^a^	3.56^a^	7.39^a^
	3.5 kGy	58.98^a^	4.28^a^	7.57^a^
**7**	Control	59.60^a^	0.83^b^	9.25^b^
	1 kGy	60.35^a^	2.31^a^	8.13^ab^
	1.5 kGy	59.34^a^	3.36^a^	7.21^a^
	2.5 kGy	57.38^a^	3.71^a^	6.91^a^
	3.5 kGy	59.05^a^	3.54^a^	6.41^a^
**27**	Control	63.90^b^	1.00^b^	7.70^a^
	1 kGy	59.68^a^	1.21^b^	7.06^a^
	1.5 kGy	61.33^a^	2.92^a^	8.87^a^
	2.5 kGy	58.92^a^	2.69^a^	8.83^a^
	3.5 kGy	58.93^a^	2.10^ab^	9.55^a^

## Section for review

Processing and products.

## Conflict of interest

One author (Gabriela K. Betancourt-Barszcz) is employed at the company that operates the electron-beam irradiation device. All authors except for G.K. Betancourt-Barszcz declare that this research was conducted without any commercial relationships that could produce potential conflicts of interest.

## CRediT authorship contribution statement

**Pheron Collie:** Writing – review & editing, Writing – original draft, Investigation, Formal analysis, Data curation. **Blesseth McDonald:** Writing – review & editing. **Olivia Hawkins:** Formal analysis, Data curation. **Marco Reina:** Writing – review & editing, Writing – original draft. **Andrew Widmer:** Methodology, Formal analysis. **Gabriela K. Betancourt-Barszcz:** Writing – review & editing, Methodology, Conceptualization. **Aaron R. Bodie:** Writing – review & editing, Supervision, Project administration, Methodology, Investigation, Conceptualization.
